# Therapeutics

**Published:** 1876-10

**Authors:** 


### III. Therapeutics.

1. *Salicylic Acid.* Peterhausen. (*Detroit Review*, September, 1876.)

Dr. H. P. V. Petershausen, reviewing the principal facts known in regard to salicylic acid reaches the following conclusions:

1. It is an excellent antiseptic when applied *externally*, and in surgery may take the place of carbolic acid on many occasions.
2. It is probably the best antipyretic now known, quinine not excluded, when applied internally.
3. As an internal remedy it has no antiseptic properties.
4. The best form in which it is given internally is sodium salicylate.

J. S. K.

2. *Local use of Bromide of Potassium.* Coomes. (*Cincinnati Medical News*, February, 1876.)

The bromide of potassium applied to mucous surfaces, is a local anæsthetic, although this effect is secondary unless used in weak solution, say ten or fifteen grains to the ounce of water. The action of the bromide when applied to mucous surfaces in substance, or saturated solution, resembles that of caustic. Its effects upon mucous surfaces are not visible, like those of an ordinary caustic. It does not whiten the tissues, nor is its application painless, as is the case with many caustics. When applied to the schneiderian membrane or palpebral conjunctiva the pain is severe and of a hot burning character. The larynx and fauces are more tolerant of its action than the eye or nose, but the pain is similar in being associated with heat. The duration of the pain is never more than a few seconds. Applied to congested mucous surfaces, it disgorges the distended vessels and increases the secretive action of the mucous follicles.

In papillary ophthalmia, commonly called "granular lids," the results of its action are similar to those obtained from the use of muriate of ammonia. It reduces the hypertrophy, increases the amount of secretion, and allays pain. Its anæsthetic properties alone give it an advantage over the ammonia.

In the treatment of nasal catarrh, where there is a dry condition of the membrane, the bromide in powder or saturated solution is an agent of great value. Where there is hypertrophy of the membrane lining the nasal cavities, with an insufficient amount of the normal secretions, a condition met with in proliferous inflammation of the membrane, insufflations of the powdered bromide, or injections of the saturated solution produce excellent results. By its use the secretions of the membrane are increased, congestion lessened, and a marked reduction made in the hypertrophied tissues. Its immediate effects in these cases of proliferous inflammation of the nasal cavities is to relieve the patient of that sense of "stuffiness" which is most always complained of.

For the last year and a half I have relied almost entirely upon the bromide of potassium as a local agent in the treatment of throat affections. It has but rarely disappointed me. The results which I have obtained from its use in this class of diseases have been most gratifying. In cases of acute tonsillitis and pharyngitis, it matters not whether in their incipiency or in the advanced stages, a solution of the bromide of potassium, sixty grains to the ounce of water, applied with a mop, or with an atomizer every hour or two, will be found to produce well nigh complete relief. In cases of ulceration the open sore should be touched with carbolic acid or nitrate of silver. In but few cases will it be necessary to apply the escharotic a second time. Under this plan of treatment all the painful and distressing symptoms that attend such cases speedily disappear. In every instance the patients treated with the bromide expressed themselves as feeling a great relief immediately after the application of the drug. These statements have been verified by the rapid reduction of temperature in the affected part, the restoration of the functions in the mucous follicles in the vicinity, the disgorgement of the distended blood vessels, and almost an entire absence of pain during the whole course of the disease.—*Half-Yearly Compendium*, July, 1876. J. S. K

